# Amyloid‐β in mitochondrial disease: mutation in a human metallopeptidase links amyloidotic neurodegeneration with mitochondrial processing

**DOI:** 10.15252/emmm.201506050

**Published:** 2016-01-26

**Authors:** Veronika Boczonadi, Rita Horvath

**Affiliations:** ^1^ Institute of Genetic MedicineWellcome Trust Centre for Mitochondrial ResearchNewcastle UniversityNewcastle upon TyneUK

**Keywords:** Genetics, Gene Therapy & Genetic Disease, Metabolism, Neuroscience

## Abstract

There is increasing evidence that common molecular pathways in neurons are closely linked with mitochondrial function and that mitochondrial dysfunction is connected to various forms of neurodegenerative diseases. For instance, mitochondria are involved in amyloid‐β (Aβ) deposition in Alzheimer's disease, although the exact molecular pathways remain largely unknown. Brunetti *et al* (2015) in this issue of *EMBO Molecular Medicine* provide a novel link between Aβ accumulation and mitochondria. A pathogenic mutation in a Norwegian family in the mitochondrial metallopeptidase PITRM1 is found to underlie a novel mitochondrial neurodegenerative phenotype associated with Aβ accumulation.

Neurological symptoms are among the most frequent clinical manifestations of primary mitochondrial diseases (Chinnery, 2015). Certain neuronal cell types are particularly sensitive to mitochondrial dysfunction, exemplified by manifestations such as Leigh syndrome, optic atrophy, cerebellar ataxia, epilepsy and peripheral neuropathy. It was originally thought that a defect in energy production caused by abnormal mitochondrial respiratory chain enzyme function is the major pathogenic factor; however, it has become evident that other roles of mitochondria such as dynamics, trafficking, inter‐organellar communication and mitochondrial quality control are also important players in neuronal dysfunction.

Our understanding of the role of mitochondria in neurons has been also recently broadened through the discovery of mitochondrial dysfunction in various forms of adult‐onset neurodegeneration (Parkinson's disease, motor neuron disease, cerebellar ataxias, spastic paraplegias), although the exact neuronal death pathways still remain largely unknown (Schon & Przedborski, 2011).

Although patients with neurodegenerative diseases frequently present with psychiatric symptoms and cognitive dysfunction, these are not prominent features or remain unnoticed in primary mitochondrial conditions. Recent data in Alzheimer's disease (AD) suggested that mitochondria are involved in amyloid‐β (Aβ) deposition, which results in neuronal toxicity.

AD is characterized by the accumulation of extracellular plaques composed mainly of Aβ and of intracellular neurofibrillary tangles composed of the microtubule‐associated protein tau. Other features include aberrant phospholipid, cholesterol and calcium metabolism and altered mitochondrial function; the underlying mechanism(s) that might explain these observations are, however, currently unknown. It has been shown that presenilin‐1, presenilin‐2, and γ‐secretase, which process the amyloid precursor protein (APP) to generate Aβ, are located predominantly in a specialized subcompartment of the endoplasmic reticulum (ER) that is physically and biochemically connected to mitochondria, referred to as mitochondria‐associated ER membranes (MAM) (Schon & Area‐Gomez, 2013). MAM function and ER–mitochondrial connectivity are significantly increased in AD, and it has been hypothesized that AD might be an ER–mitochondrial communication disorder (the “MAM hypothesis”) (Schon & Area‐Gomez, 2013).

Progressive accumulation of Aβ in mitochondria is associated with diminished enzymatic activity of respiratory chain complexes III and IV and a reduction in the rate of oxygen consumption. The formation of mitochondrial Aβ plaques precedes the characteristic extensive extracellular Aβ deposition and is evident before the onset of clinical symptoms both in AD patients and in APP transgenic mice, where they are detected as early as 4 months of age (Lustbader *et al*, 2004; Manczak *et al*, 2006). Aβ interacts with alcohol dehydrogenase (ABAD) in the mitochondria and prevents nicotinamide adenine dinucleotide (NAD) binding, leading to exaggerated neuronal oxidative stress and impaired memory. These data suggest that the ABAD–Aβ interaction may be a therapeutic target in AD (Lustbader *et al*, 2004). However, there are many open questions on the mechanisms and specifically how intracellular Aβ impairs cellular functions leading to neuronal impairment.

One possibility is that Aβ accumulation via the outer membrane transporter (TOM) affects mitochondrial functions not only by blocking the entry of other cytosolic proteins but also by interfering with multiple processes including oxidative phosphorylation, reactive oxygen species (ROS) production, mitochondrial dynamics and interaction with other mitochondrial proteins (Pagani & Eckert, 2011). Furthermore, Aβ was mainly associated with synaptic mitochondria in the cortical neurons of APP/Aβ mice, highlighting the importance of mitochondrial dysfunction in AD (Du *et al*, 2010).

The work by Brunetti *et al* (2015) in this issue of *EMBO Molecular Medicine* provides a novel link between Aβ accumulation and mitochondria. Intracellular degradation of Aβ is performed by two enzymes of the pitrilysin oligopeptidase family: the human insulin‐degrading enzyme (IDE) and a mitochondrial‐specific pitrilysin metallopeptidase 1 (PITRM1), which also digests the mitochondrial targeting sequences (MTS) of imported proteins across the inner mitochondrial membrane (Falkevall *et al*, 2006). Decreased PITRM1 proteolytic activity contributes to Aβ accumulation in the mitochondria, leading to neuronal death that is exacerbated in AD (Alikhani *et al*, 2011). Brunetti *et al* (2015) now identify a homozygous *PITRM1* mutation (c.548G>A, p.Arg183Gln) in two siblings from an isolated population in Norway, presenting with mild intellectual disability, spinocerebellar ataxia, cognitive decline and psychosis by homozygosity mapping and whole exome sequencing. In support of the pathogenic role of the mutation, the authors performed a meticulous set of experiments including clinical investigations, studies in fibroblasts and skeletal muscle of patients and validating the results in a yeast model and in *Pitrm1* ^+/−^ mice. The clinical picture of the patients was unusual for mitochondrial disease with ataxia, cognitive and psychiatric problems from childhood, showing very slow progression until their late sixties. Brain imaging revealed cerebellar and mild cerebral atrophy and unilateral signal changes in the thalamus in one of the siblings. Biochemical activities of all respiratory chain enzymes, as well as citrate synthase, were decreased. The homozygous missense mutation resulted in decreased level of the PITRM1 protein, severe growth defect in galactose medium and decreased mitochondrial membrane potential in patient fibroblasts. The authors modelled the mutation in Saccharomyces cerevisiae (*cym1* ^*R163Q*^), which provided convincing evidence that the mutation resulted in the incomplete digestion of Aβ in the mitochondria. Finally, heterozygous *Pitrm1* ^*+/*−^ mice replicated several neurological symptoms seen in patients and showed an age‐dependent Aβ accumulation in the brain, similar to AD amyloid plaques. Furthermore, impaired Pitrm1 activity in *Pitrm1* ^+/−^ mice led to the mitochondrial accumulation of MTS species affecting the maturation and function of imported mitochondria proteins.

The data demonstrate that partial impairment of the metallopeptidase PITRM1 within the mitochondria causes a novel mitochondrial disease with neurodegeneration, which is associated with the accumulation of APP and Aβ_1–42_, directly linking abnormal mitochondrial proteostasis with Aβ accumulation (Fig 1). However, there are some limitations of the study, since Aβ deposition has been shown in a mouse model, but not in the patients with mutant *PITRM1,* and additional patients with similar clinical presentation and a different mutation in the same gene are still lacking. Identification of variants in *PITRM1* or its mitochondrial interacting partners within AD cohorts may further strengthen the link between mitochondria and neurodegeneration. Exploring the pathology of cortical neurons in patients with various mitochondrial diseases and in ageing may identify further roles of mitochondria in cognition. Characterization of the molecular mechanism of mitochondrial dysfunction in neuronal cell death will likely have further implications for our understanding of the pathophysiology and designing treatment for adult‐onset neurodegenerative conditions.

**Figure 1:**
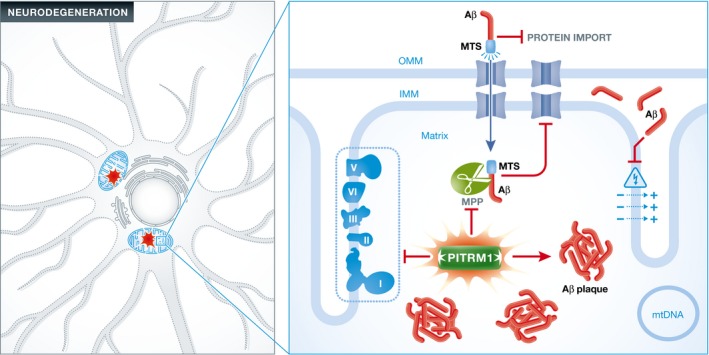
Impaired amyloid‐β (Aβ) degradation due to the decreased activity of pitrilysin metallopeptidase 1 (PITRM1) contributes to Aβ accumulation in the mitochondria, leading to neuronal death The presence of Aβ within the mitochondria affects several aspects of mitochondrial function. Interaction of Aβ with the outer mitochondrial membrane (OMM) impairs the transport of nuclear‐encoded mitochondrial proteins, such as subunits of the respiratory chain complexes, into the organelle via the translocase of the outer membrane (TOM) import machinery. Insufficient PITRM1 impairs the degradation of mitochondrial targeting sequences (MTS) cleaved from the imported proteins by the mitochondrial processing peptidase (MPP), further disturbing the import of proteins into the mitochondria. Respiratory chain dysfunction leads to decreased ATP synthesis in addition to increased reactive oxygen species (ROS) production. Finally, Aβ reduces proton translocation from the matrix to the intermembrane space, thus impairing the mitochondrial membrane potential (MMP).
